# Effect of salinity stress and surfactant treatment with zinc and boron on morpho-physiological and biochemical indices of fenugreek (*Trigonella foenum-graecum*)

**DOI:** 10.1186/s12870-024-04800-7

**Published:** 2024-02-27

**Authors:** Atika Khan, Safura Bibi, Talha Javed, Athar Mahmood, Shahid Mehmood, Muhammad Mansoor Javaid, Baber Ali, Muhammad Yasin, Zain Ul Abidin, Mohammad Khalid Al-Sadoon, Babar Hussain Babar, Rashid Iqbal, Tabarak Malik

**Affiliations:** 1 Department of Botany, Faculty of Sciences, University of Agriculture Faisalabad, Faisalabad, 38040 Pakistan; 2 Institute of Tropical Bioscience and Biotechnology, Chinese Academy of Tropical Agricultural Sciences, Haikou-571101, China; 3 Department of Agronomy, University of Agriculture Faisalabad, Faisalabad, 38040 Pakistan; 4 Biotechnology Research Institute, Chinese Academy of Agricultural Sciences, Beijing, 100081 China; 5 Department of Agronomy, College of Agriculture, University of Sargodha, Sargodha, PK-40100 Pakistan; 6 Department of Plant Sciences, Quaid-i-Azam University, Islamabad, 45320 Pakistan; 7 Department of Agricultural Sciences, Faculty of Sciences, Allama Iqbal Open University Islamabad, Islamabad, Pakistan; 8 Department of Zoology, College of Science, King Saud University, PO BOX 2455, Riyadh, 11451 Saudi Arabia; 9 Vegetable Section, Ayub Agricultural Research Institute, Faisalabad, Pakistan; 10 Department of Agronomy, Faculty of Agriculture and Environment, The Islamia University of Bahawalpur Pakistan, Bahawalpur, 63100 Pakistan; 11 Department of Biomedical Sciences, Institute of Health, Jimma University, Jimma, Ethiopia 378

**Keywords:** Boron, Zinc, Chlorophyll *a*, Total soluble proteins, Sodium

## Abstract

Micronutrient application has a crucial role in mitigating salinity stress in crop plants. This study was carried out to investigate the effect of zinc (Zn) and boron (B) as foliar applications on fenugreek growth and physiology under salt stress (0 and 120 mM). After 35 days of salt treatments, three levels of zinc (0, 50, and 100 ppm) and two levels of boron (0 and 2 ppm) were applied as a foliar application. Salinity significantly reduced root length (72.7%) and shoot length (33.9%), plant height (36%), leaf area (37%), root fresh weight (48%) and shoot fresh weight (75%), root dry weight (80%) and shoot dry weight (67%), photosynthetic pigments (78%), number of branches (50%), and seeds per pod (56%). Fenugreek’s growth and physiology were improved by foliar spray of zinc and boron, which increased the length of the shoot (6%) and root length (2%), fresh root weight (18%), and dry root weight (8%), and chlorophyll a (1%), chlorophyll b (25%), total soluble protein content (3%), shoot calcium (9%) and potassium (5%) contents by significantly decreasing sodium ion (11%) content. Moreover, 100 ppm of Zn and 2 ppm of B enhanced the growth and physiology of fenugreek by reducing the effect of salt stress. Overall, boron and zinc foliar spray is suggested for improvement in fenugreek growth under salinity stress.

## Introduction

The world’s oldest medicinal plant is fenugreek, and it belongs to the legume family. It was originally grown as a pasture crop; however, fenugreek is mostly grown as a spice crop [1]. Fenugreek has pharmacological importance because of secondary metabolites such as flavonoid, terpenoids, pyridine-type alkaloids (trigonelline and choline) and protein (arginine, histidine, and lysine) [2]. It is cultivated mostly in China, Pakistan, India, and Canada. In India, fenugreek production is about 55 thousand tons per year. Due to these secondary metabolites’ fenugreek plant is used as anti-diabetic, digestive stimulant, cardio, and gastro protective. It has proteins (26%), fats (7%), and carbohydrates (58%) [3].

In Pakistan fenugreek also known as Methi and its production is mainly affected by salt stress because it belongs to leguminous family as higher concentration of salts in soil causes reduction in root hairs and rhizobia formation on roots [4]. Plants are affected by several types of environmental conditions like biotic and abiotic stresses [5–7]. These conditions have negative effects on morphology, physiology as well as production of plants [8, 9].

Salt stress is global issue which affects about 20% of agricultural area and reduce crop yield [10]. Among the many salts found in the soil, NaCl is the most common [10, 11]. This stress can be represented as oxidative stress in plants due to the production of reactive species of oxygen or nitrogen in higher concentration [12]. Salinity has negative impacts on physiology and metabolic activities of plants [13]. Symptoms of these changes include decreased leaf area, maximum leaf thickness and succulence, abscission of leaves, root and shoot necrosis, and shorter internode length [14, 15]. Salinity currently affects 7% of land, and it is expected that by 2050, salinity will affect 50% of soil [16, 17]. Salt stress have negative effect on growth and yield of plants []. Plant roots, shoots, leaves, flowers, and all other parts can be used as a medicine. Fenugreek has secondary metabolic properties which are present in these parts, but growth of these plant parts is retarded when they face any kind of stress like salinity. Similarly, saline soil cause reduction in active chemical contents of fenugreek plant [18, 19].

To cope with oxidative stress different types of micronutrients are used as foliar [] or soil application in plants [20]. Generally, several types of approaches have been made to mitigate salinity stress and nutrient application is one of them. Micronutrients application can be helpful for the improvement of plants under stressed condition [21, 22]. These nutrients include iron, manganese, zinc, cobalt, boron, nitrogen [23] and their foliar spray improves the physiology, growth, and production of plant [24]. Zinc is colorless micronutrient and it act as a synthetic antioxidant as well as it has key role in the development of structure and enzyme catalysis [25]. Zinc plays a vital role in the formation of auxin in plants which is helpful in promoting growth. It is involved in controlling water absorption in plants [26]. Zinc nano oxides foliar application is used to enhance secondary metabolites production in plants [27].

Numerous metabolic activities in plants, including lignification, transport of sugar, structure of cell wall, membrane permeability, protein and carbohydrate metabolism, and nucleic acid synthesis are regulated by boron application [28]. Boron reduces the IAA oxidase activity resulting in increased auxin content. It is involved in the nucleic acid formation in plants [29]. It has been reported that foliar application of zinc with boron is helpful in enhancing the growth of plant [30]. Boron is also a micronutrient in plants which is required in narrow range for proper growth, but its higher concentration can be toxic. It not only enhances the yield but also improves the quality of crops. Pollen tube germination and growth is increased through boron application which enhances the reproductive process of plants [31]. Under low temperature conditions, when there is deficiency of boron then nutrients uptake by plant roots is decreased which results in retarded growth [32]. As a result, there isn’t a single study that examines how different micronutrients respond in comparison as a fenugreek limiting factor [33]. Therefore, this experiment was conducted to examine the impact of zinc and boron as foliar spray to mitigate the effect of salinity on fenugreek plant growth, yield, physiology, and ions.

## Materials and methods

### Experimental site and plant materials

This research was performed at UAF Postgraduate Agricultural Research Station (Latitude: 31.383721 and Longitude: 72.989998) in plastic pots [width (24 cm) and depth (30 cm)] separately containing 10 kg dry river sand, which was thoroughly washed. Plant seeds of one variety of fenugreek known as “Desi Methi” were collected from the Ayub Agricultural Research Center, Faisalabad.

### Experimental design and treatments

Sand-filled plastic pots 30 cm diameter and 25 cm height in dimension were laid in a completely randomized design (CRD) in a factorial arrangement with three replications for each treatment. At equal depth and distance, seeds were sown. Plants were applied with Hoagland solution at three week old plant stage and two levels of salt (0, 120mM NaCl), were applied to each pot. The salt concentration of 120 mM was maintained in aliquot parts of 60 mM to prevent salt shock. Sand was watered with saline water regularly twice a week till the termination of experiment and NaCl applications (0, 120 mM) were given together with water. Plants were irrigated until saturated, with the excess solution allowed to drain into collection pots. Foliar application of zinc (0, 50ppm, 100ppm) and boron (0, 2ppm) concentration was sprayed on three weeks old plants. Tween-20 (0.1%) was applied as a surfactant to enhance the absorbance of the solution. The fenugreek plants in each pot provided 20 ml of each level of zinc concentration (0, 50ppm, 100ppm) and boron (0, 2ppm) soon after sunset to prevent solution evaporation. After 21 days of foliar spray, three plants were carefully uprooted from each pot for data collection of shoots and roots.

### Growth

Three plants from each treatment were harvested to evaluate morphological parameters, including height of plant, length of shoot, fresh weight of shoot, dry weight of shoot, length of root, fresh weight of root, dry weight of root, leaf area, no. of branches, no. of seeds, no. of leaves and weight of 100 seeds.

### Physiology

#### Chlorophyll content

Chlorophyll and carotenoid pigments were determined by [34] and [35] methods in leaves. A 0.5 g of fresh leaves was powdered and 5 ml of acetone (80%) was added in it. The extract was centrifuged and absorbance at different wavelength (645 and 663 nm) was measured by spectrophotometer. Values were calculated by these formulas.

$$\eqalign{{\rm{Chl}}{\rm{.}}\,a\,\left( {{\rm{mg}}\,{{\rm{g}}^{ - {\rm{1}}}}\,{\rm{FW}}} \right)\,{\rm{ = }} & \,\left[ {{\rm{12}}{\rm{.7}}\,\left( {{\rm{OD663}}} \right)\, - \,{\rm{2}}{\rm{.69}}\,\left( {{\rm{OD645}}} \right)} \right] \cr & {\rm{ \times }}\,{\rm{V}}\,{\rm{/}}\,{\rm{1000}}\,{\rm{ \times }}\,{\rm{W}} \cr}$$

$$\eqalign{{\rm{Chl}}{\rm{.}}\,b\,\left( {{\rm{mg}}\,{{\rm{g}}^{ - {\rm{1}}}}\,{\rm{FW}}} \right)\,{\rm{ = }} & \,\left[ {{\rm{22}}{\rm{.9}}\,\left( {{\rm{OD645}}} \right)\, - \,{\rm{4}}{\rm{.68}}\,\left( {{\rm{OD663}}} \right)} \right] \cr & {\rm{ \times }}\,{\rm{V}}\,{\rm{/}}\,{\rm{1000}}\,{\rm{ \times }}\,{\rm{W}} \cr}$$

$$\eqalign{{\rm{Carotenoids}}\,\left( {{\rm{mg}}\,{{\rm{g}}^{ - {\rm{1}}}}\,{\rm{FW}}} \right)\,{\rm{ = }} & \,\left[ {{\rm{4}}{\rm{.16}}\,\left( {{\rm{OD480}}} \right)\, - \,{\rm{0}}{\rm{.89}}\,\left( {{\rm{OD663}}} \right)} \right] \cr & {\rm{ \times }}\,{\rm{V}}\,{\rm{/}}\,{\rm{1000}}\,{\rm{ \times }}\,{\rm{W}} \cr}$$

#### Total protein content (mg g^− 1^ FW)

Proteins were estimated using [36] method. Leaf sample 0.5 g was grinded and phosphate buffer 10mL of 0.02 M having pH 7 was added and extract was filtered. 1mL of plant sample and 1mL of extract was taken in test tubes and kept at room temperature for 30 min. Then, Folin reagent 0.5mL was added. Again, these materials were stored at ambient temperature and 5mL distilled water was added. Absorbance was taken at 620 nm. Buffer (1mL) and Brad Ford (2 mL) was taken as a blank.

### Nutrients analysis for shoot

Acid digestion is used for the determination of mineral ions by following [37]. Dried shoot material (0.1 g) was powdered and mixed with 2 ml of conc. H_2_SO_4_ in digestion flasks for digestion. Then 4ml H_2_O_2_ was added in it and heated at 350 °C. Heating process is continued until the fumes produce. Then again 1mL of 35% H_2_O_2_ was added and continued the process until mixture be-comes colorless. Then mixture is diluted by adding distilled water up to 50 ml and filtered. Mineral ions like Na^+^, K^+^, and Ca^2+^ were identified using a flame photometer.

### Salt tolerance index

The salt tolerance index (STI) was determined by comparing the total dry weight of plants obtained from 100 seeds grown under varying salt concentrations to the total dry weight of plants obtained under normal conditions (control). The formula for calculating the STI is as follows.,

Salt Tolerance Index = (Total Dry Weight at salinity / Total Dry Weight in control) x 100.

### Statistical analysis

Data of all parameters was analyzed using factorial completely randomized design (CRD) with three replications by using software COSTAT [38] and means of treatment were compared by using Tukey test at 5% level of significance. Pearson correlation analysis was performed using R Studio Version 4.1.2.

## Results

### Growth

**Fig. 1:**
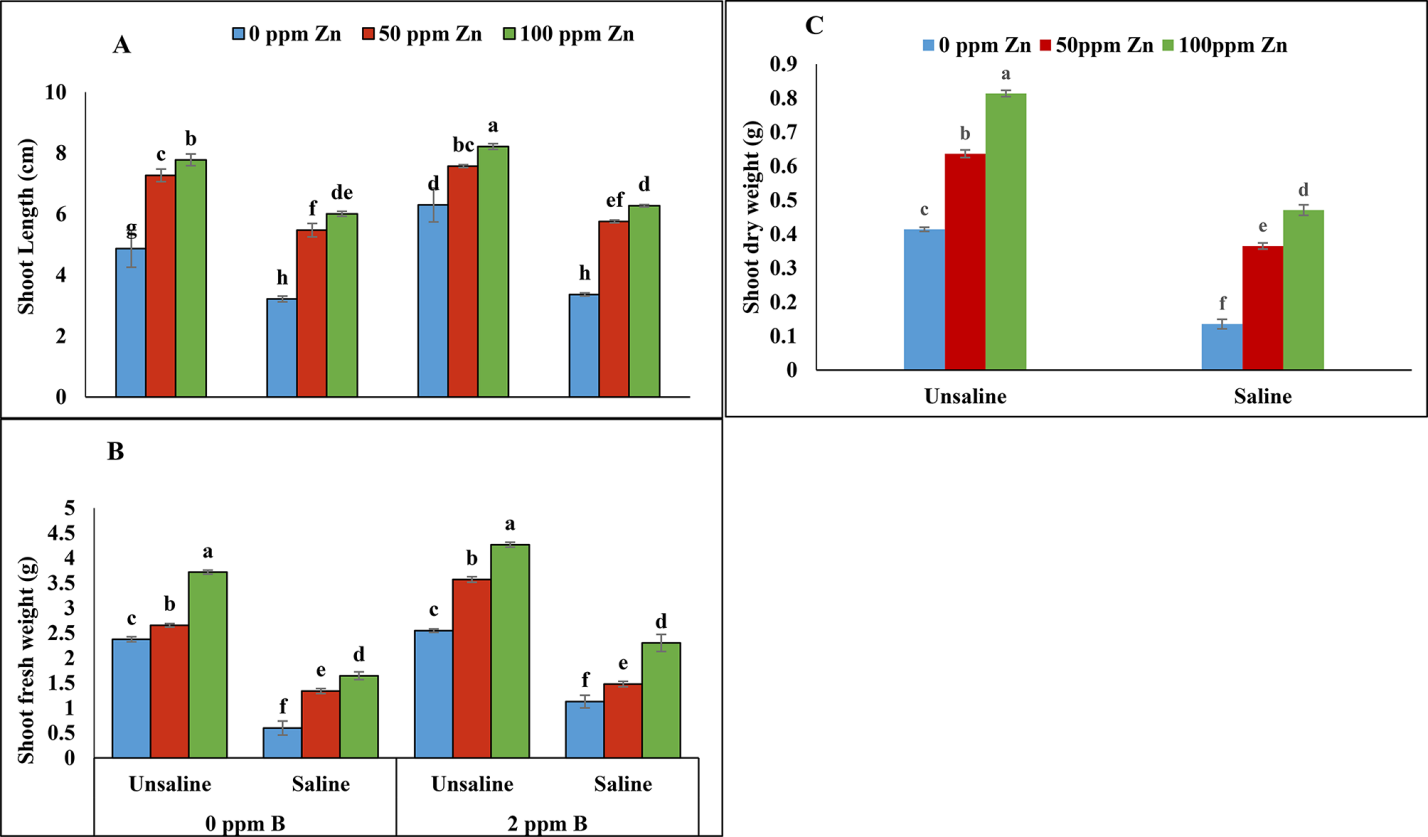
Effect of foliar application of zinc and boron on shoot length **(A)**, shoot fresh weight **(B)** and shoot dry weight **(C)** of fenugreek grown under salinity stress Values represent means ± S.E

According to the analysis of variance salinity interaction with zinc and zinc with boron showed non-significant results while significant (*P* ≤ 0.05) results were obtained from salinity interaction with zinc and boron in combination and salinity with boron regarding shoot length (Table 1; Fig. 1A). A significant reduction in shoot length was observed under salinity level of 120mM. However, foliar application of zinc at 50 ppm and 100ppm level under un-saline and saline condition improved shoot length. Similarly, foliar application of boron (2ppm) significantly increased shoot length in both un-saline and saline condition. Foliar application of zinc (100ppm) and boron (2ppm) enhanced shoot length to maximum value under un-saline conditions. Similarly, maximum reduction was noted under saline conditions at 0 ppm level of boron and zinc.

**Table 1:** Mean square values for morphological and yield parameters of fenugreek grown under salinity stress with foliar application of zinc and boron

SOV-	Df	RL	SL	PH	LA	NO. of leaves	RFW	RDW	SFW	SDW	No. of branches/ plant	No. of seeds/pod	100 SW
Salinity	1	45.38**	35.46**	107.47**	0.08**	4923.36**	0.22**	0.06**	28.34**	0.95**	336.11**	160.44**	1.96**
Zn	2	18.74**	23.14**	105.80**	0.00**	3259.03**	0.45**	0.02**	5.25**	0.39**	196.08**	121.03**	1.52**
B	1	3.54**	2.05**	19.36**	4.10**	552.25**	0.10**	0.00**	2.20**	0.09**	49.00**	16.00**	0.33**
Salinity × Zn	2	2.91**	0.22ns	1.44*	0.00**	212.69**	0.01*	6.80ns	0.14**	0.01**	11.36**	6.03**	0.06**
Salinity × B	1	0.20ns	0.55*	0.02ns	1.92**	23.36ns	0.00ns	1.25ns	0.02ns	0.01**	4.00*	0.44ns	0.02*
Zn × B	2	0.91**	0.22ns	0.04ns	5.63ns	3.58ns	5.83ns	4.30ns	0.05*	8.36*	3.25**	0.08ns	0.00ns
Salinity×Zn×B	2	0.04ns	0.36*	0.30ns	1.03ns	0.36ns	1.02ns	3.08*	0.26**	3.66ns	2.08*	0.19ns	0.00ns
Error	24	0.07	0.07	0.31	8.20	8.11	0.00	6.13	0.01	1.21	0.56	0.31	0.35
LSD Salinity		0.183	0.182	0.38	0.051	1.95	0.024	0.0053	0.059	0.0075	0.51	0.38	0.046
LSD B		0.183	0.182	0.38	0.051	1.95	0.024	0.0053	0.059	0.0075	0.51	0.38	0.046
LSD Zn		0.224	0.223	0.46	0.072	2.39	0.029	0.0065	0.072	0.0092	0.62	0.46	0.057

ns; non-significant, SOV; sum of variance, Zn; zinc, B; boron, Df; degree of freedom, RL; root length, SL; shoot length, PH; plant height, LA; Leaf area, RFW; root fresh weight, RDW; root dry weight, SFW; shoot fresh weight, SDW; shoot dry weight, SW; seed weight

** Significant at *P* ≤ 0.05

Similar trend was observed for shoot fresh and dry weight. Significant results (*P* ≤ 0.05) were obtained for parameters on foliar application of zinc and boron under salinity. Salinity decreased fresh and dry weight of fenugreek shoot while foliar application of zinc with boron increased shoot dry weight non significantly under salt condition (120mM NaCl). Similarly, foliar application of zinc with boron enhanced shoot fresh weight significantly (Table 1; Fig. 1B and C). Maximum fresh and dry weight was noted at 100ppm of zinc and 2 ppm of boron under un-saline conditions. However, minimum fresh and dry weight of fenugreek shoot were recorded at boron (0ppm) and zinc (0ppm) under saline condition.

**Fig. 2:**
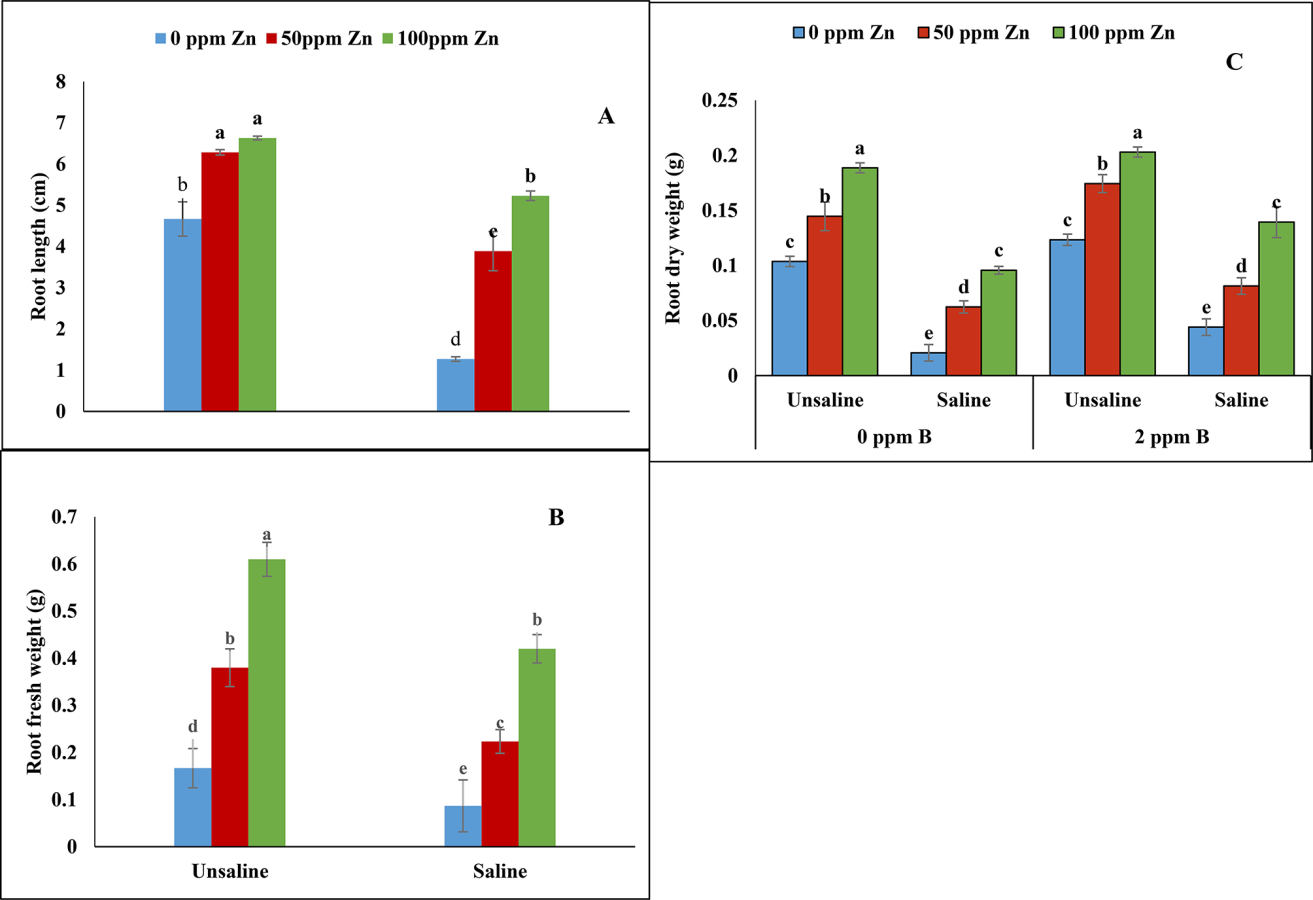
Effect of foliar application of zinc and boron on root length **(A)**, root fresh weight **(B)** and root dry weight **(C)** of fenugreek grown under salinity stress. Values represent means ± S.E

According to the analysis of variance root length showed non-significant results under salinity and foliar application of zinc with boron (Table 1; Fig. 2A). However, significant results were obtained from foliar application of zinc and boron singly with salinity (*P* ≤ 0.05). Salinity significantly reduced root length which was increased by foliar application of zinc and boron. It was noted that 100 ppm of zinc application without boron resulted in reduction of root length under saline condition. Maximum root length was followed by the foliar application of zinc (100 ppm) with boron (2 ppm) under un-saline conditions. Similarly, minimum root length was observed at 0ppm zinc and 0ppm boron under saline conditions.

**Fig. 3:**
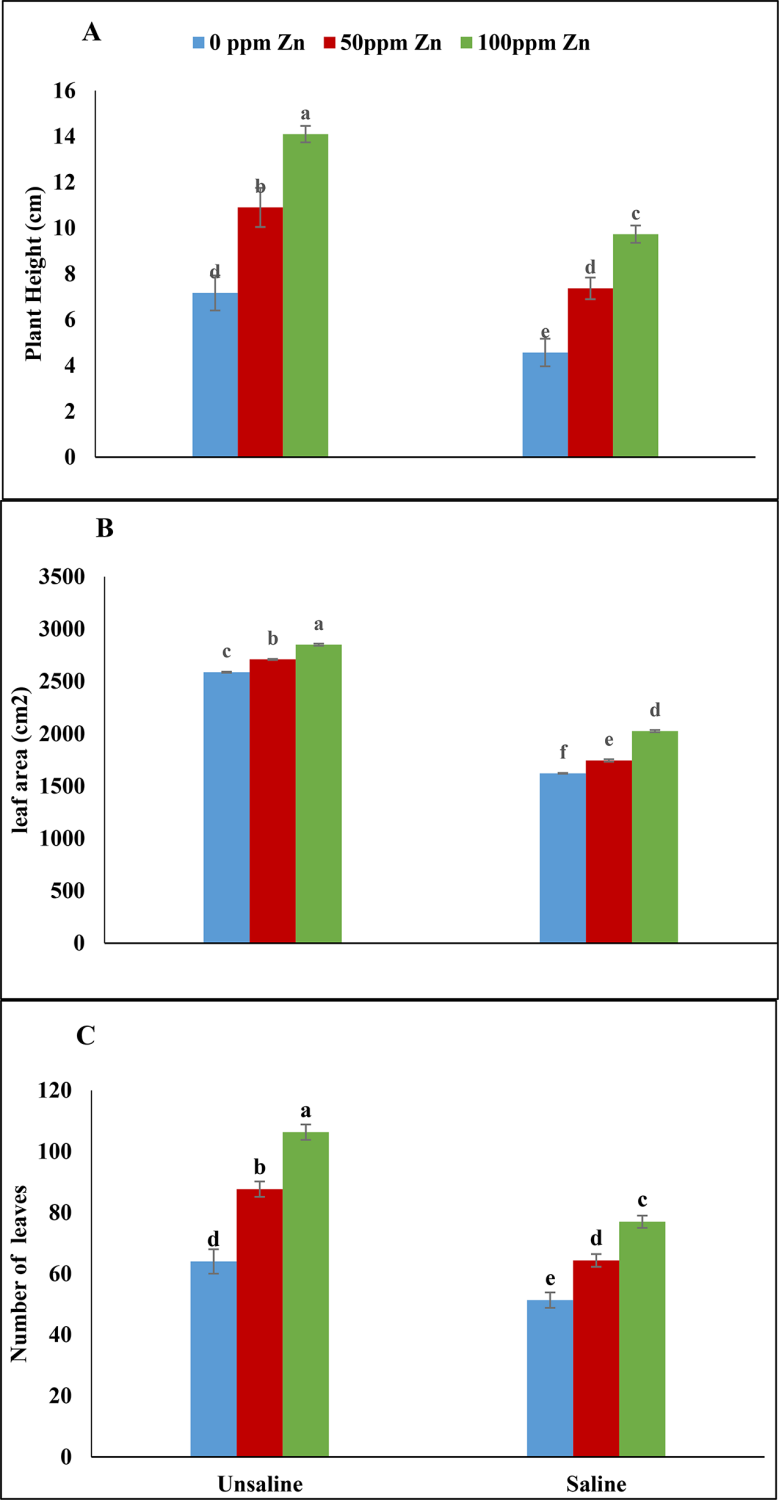
Effect of foliar application of zinc and boron on plant height **(A)**, leaf area **(B)** and number of leaves **(C)** of fenugreek grown under salinity stress. Values represent means ± S.E

Non-significant results were obtained for root fresh weight, plant height and number of leaves under saline condition and foliar application of zinc with boron. However, salinity and foliar application of zinc with boron showed significant results for root dry weight and leaf area. Maximum reduction in root fresh and dry weight was observed at 0 ppm zinc and 0ppm boron under saline condition. The foliar application of zinc and boron together under saline and un-saline conditions increased root fresh weight non significantly while significant results were obtained for root dry weight. Same trend was recorded for plant height, number of leaves and leaf area. Salinity reduced plant height and number of leaves of fenugreek plant non significantly while leaf area was significantly reduced. Similarly, maximum plant height, number of leaves and leaf area were recorded in foliar application of zinc and boron together under un-saline conditions (Table 1; Figs. 2B and C and 3A and C).

### Yield

**Fig. 4:**
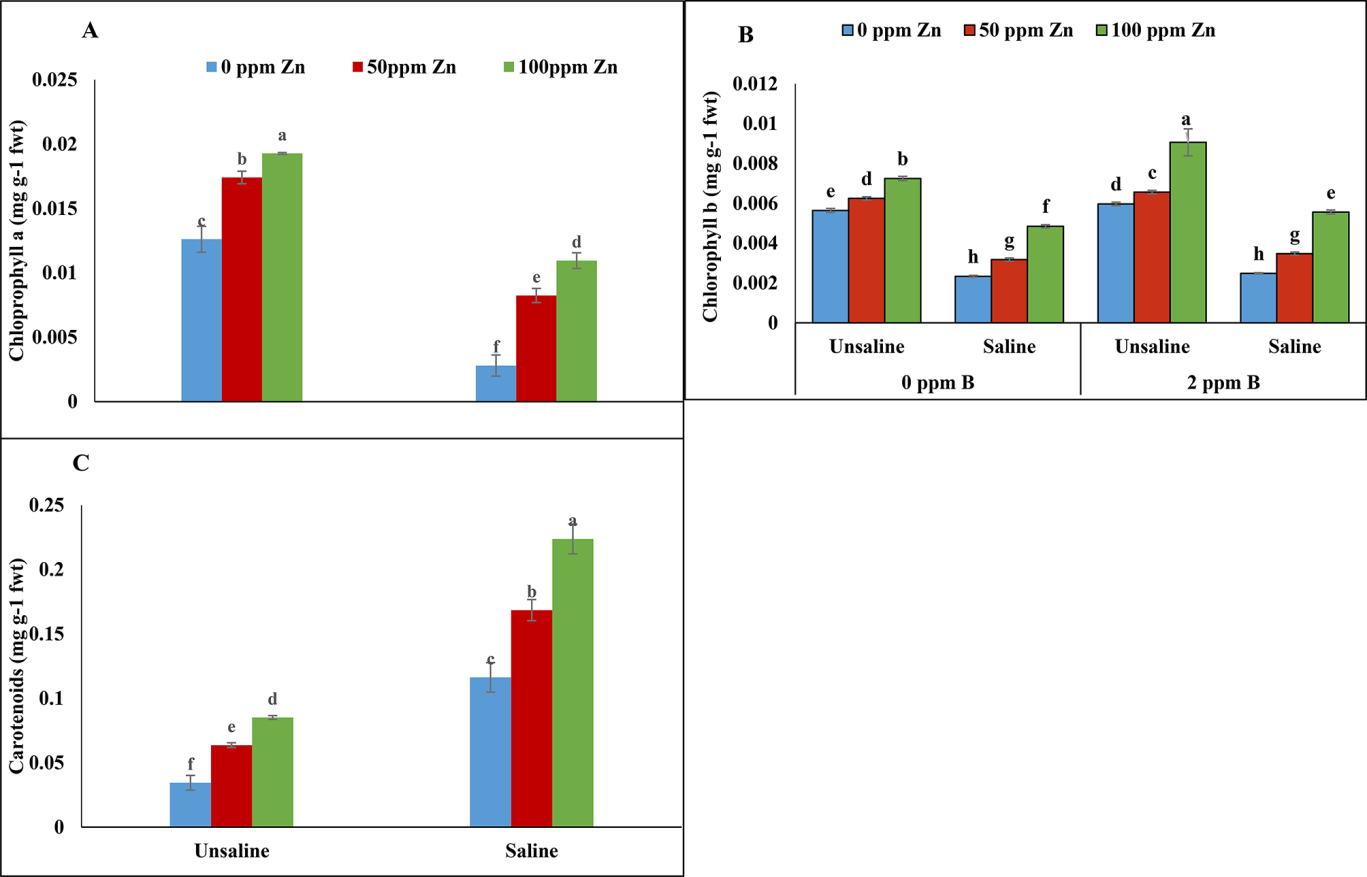
Effect of foliar application of zinc and boron on chlorophyll *a***(A)**, chlorophyll *b***(B)** and carotenoids **(C)** of fenugreek grown under salinity stress. Values represent means ± S.E. Significant differences among row spacing were measured by the least significant difference (LSD) at *p* > 0.05 and indicated by different letters

According to the analysis of variance salinity and micronutrients (zinc and boron) application showed significant results for number of branches. Salinity reduced number of branches of fenugreek plants treated with zinc and boron (0ppm) application. However, foliar application of zinc with boron (2ppm) increased the number of branches under saline and un-saline condition. Application of zinc (100ppm) and boron (2ppm) increased number of branches to maximum value under un-saline conditions (Table 1; Fig. 4A). Non-significant results were obtained for the number of seeds per pod and 100 seed weight on zinc and boron (0ppm) application under salinity. Salinity decreased the number of seeds and seed weight which was improved by foliar application of zinc and boron. Maximum number of seeds and seed weight was recorded at 100ppm of zinc and 2ppm of boron application under un-saline conditions. Foliar application of zinc (100ppm) and boron (0ppm) under saline condition resulted in reduction of seed weight and number of seeds (Table 1; Fig. 4B-C).

### Physiology

#### Chlorophyll content

**Fig. 5:**
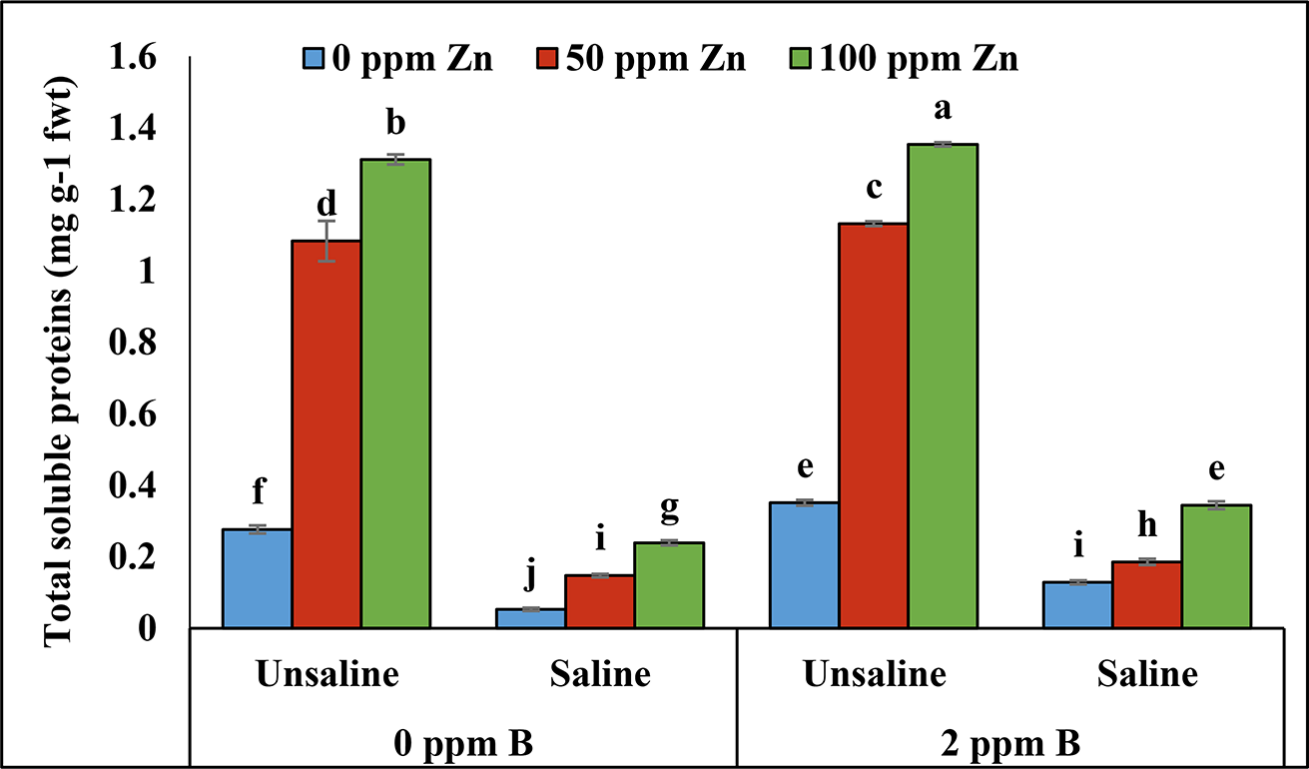
Effect of foliar application of zinc and boron on total soluble proteins of fenugreek grown under salinity stress. Values represent means ± S.E. Significant differences among row spacing were measured by the least significant difference (LSD) at *p* > 0.05 and indicated by different letters

According to analysis of variance chlorophyll *a* showed non-significant result on micronutrients application under salinity. Foliar application of zinc (100 ppm) and boron (2ppm) enhanced chlorophyll *a* under saline and un-saline condition. Similarly, zinc and boron (0ppm) improved chlorophyll *a* value under salinity. However, salinity and foliar application of micronutrient (zinc and boron) showed significant results for chlorophyll *b*. Application of zinc (100ppm) and boron (0ppm) decreased chlorophyll *b* content under saline conditions. Maximum chl. *a*, and chl. *b* values were recorded at zinc (100ppm) and boron (2ppm) application. Minimum chlorophyll concentration was recorded at 0ppm zinc and 0ppm boron under saline conditions (Table 2; Fig. 5A-B).

**Table 2:** Mean square values for physiological and biochemical parameters of fenugreek grown under salinity stress with foliar application of zinc and boron

SOV	Df	Chl a	Chl b	Car	Na^+^	K^+^	Ca^2+^	STI	TSP
**Salinity**	1	7.14**	8.88**	0.012**	423.67**	245.44**	160.44**	57031.54	4.86**
**Zn**	2	1.38**	2.10**	0.01**	219.09**	225.78**	204.53**	22754.99	1.18**
**B**	1	2.48**	3.27**	0.00**	37.01**	38.03**	28.44**	5510.61	0.04**
**Salinity × Zn**	2	5.53**	1.57*	0.00**	7.34**	2.86*	2.53*	177.18	0.60**
**Salinity × B**	1	3.64ns	4.20**	5.11*	0.00ns	1.00ns	0.03ns	182.32	0.00ns
**Zn × B**	2	2.54**	9.88**	3.1ns	0.22ns	0.44ns	0.03ns	28.92	0.00ns
**Salinity×Zn×B**	2	1.40ns	2.58**	7.19ns	0.22ns	0.75ns	0.36ns	30.10	0.00*
**Error**	24	4.49	4.49	5.48	0.51	0.49	0.51	12.53	3.29
**LSD Salinity**		4.61	1.45	0.005	0.49	0.47	0.493	2.43	0.012
**LSD B**		4.61	1.45	0.005	0.49	0.47	0.493	2.43	0.012
**LSD Zn**		5.65	1.78	0.006	0.60	0.58	0.604	2.98	0.015

**, significant at *P* ≤ 0.05, ns; non-significant, SOV; sum of variance, Df; degree of freedom, Chl a; chlorophyll a, Chl b; chlorophyll b, Car; carotenoids, STI; salt tolerance index, TSP; total soluble proteins

#### Carotenoids content

Analysis of variance revealed non-significant results for carotenoids under salinity and foliar application of zinc with boron. Maximum carotenoids content was observed in plants treated with zinc (100 ppm) and boron (2ppm) under salinity. However, zinc and boron under un-saline conditions showed a little effect on carotenoids content. Minimum carotenoids value was observed at 0ppm zinc and 0ppm boron under un-saline conditions (Table 2; Fig. 5C).

#### Total soluble protein content (TSP)

**Fig. 6:**
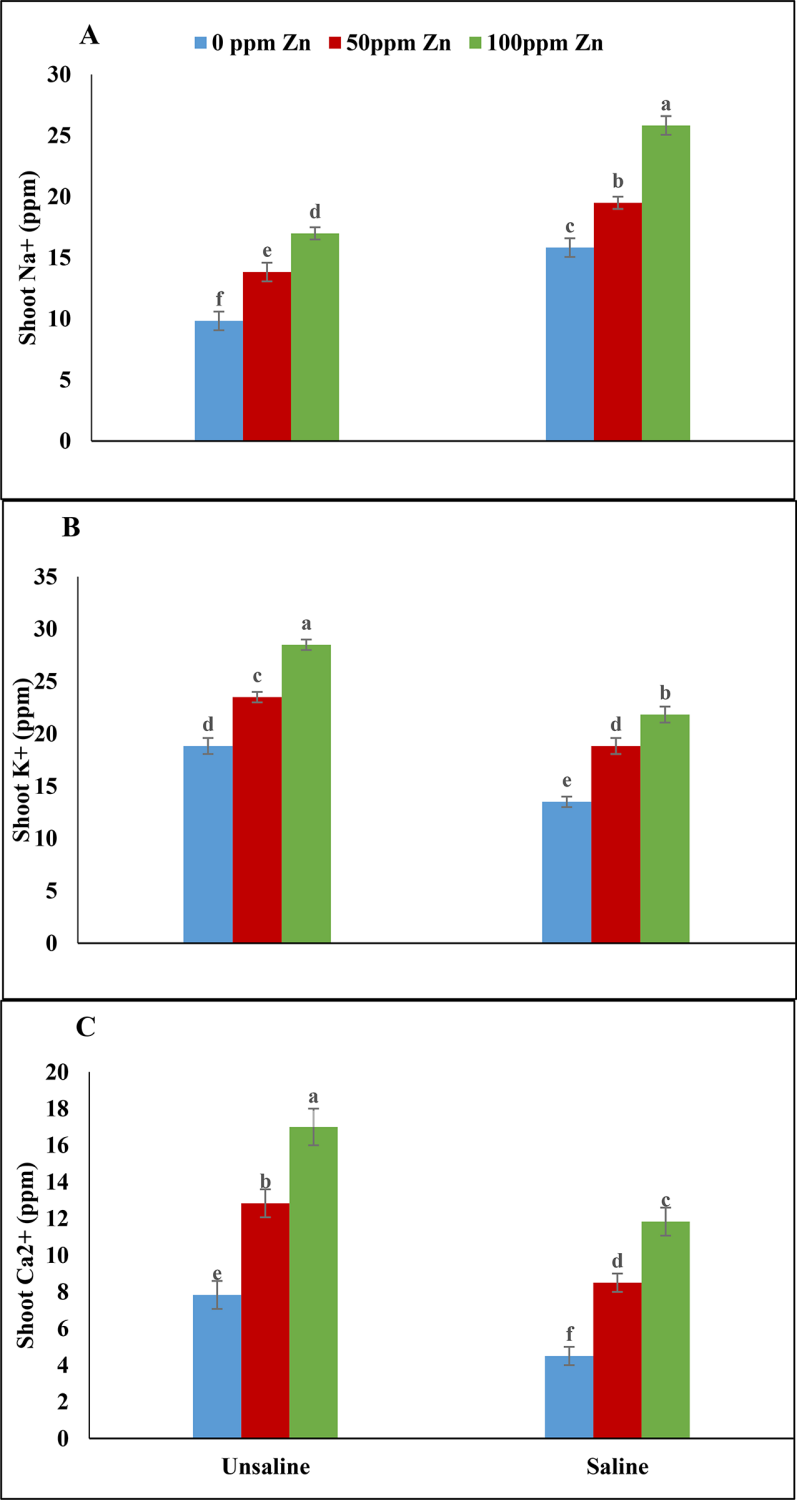
Effect of foliar application of zinc and boron on shoot Na^+^**(A)**, shoot K^+^**(B)**, and shoot Ca^2+^ content of fenugreek grown under salinity stress. Values represent means ± S.E. Significant differences among row spacing were measured by the least significant difference (LSD) at *p* > 0.05 and indicated by different letters

According to analysis of variance salinity and foliar application of micronutrients showed significant results in total soluble protein content. Salinity highly reduced total soluble protein content. Application of zinc (100ppm) and boron (2ppm) maximized total soluble proteins under un-saline condition. Minimum proteins content was recorded at 0ppm of zinc and 0ppm of boron under saline conditions (Table 2; Fig. 6).

### Nutrients analysis for shoot

**Fig. 7:**
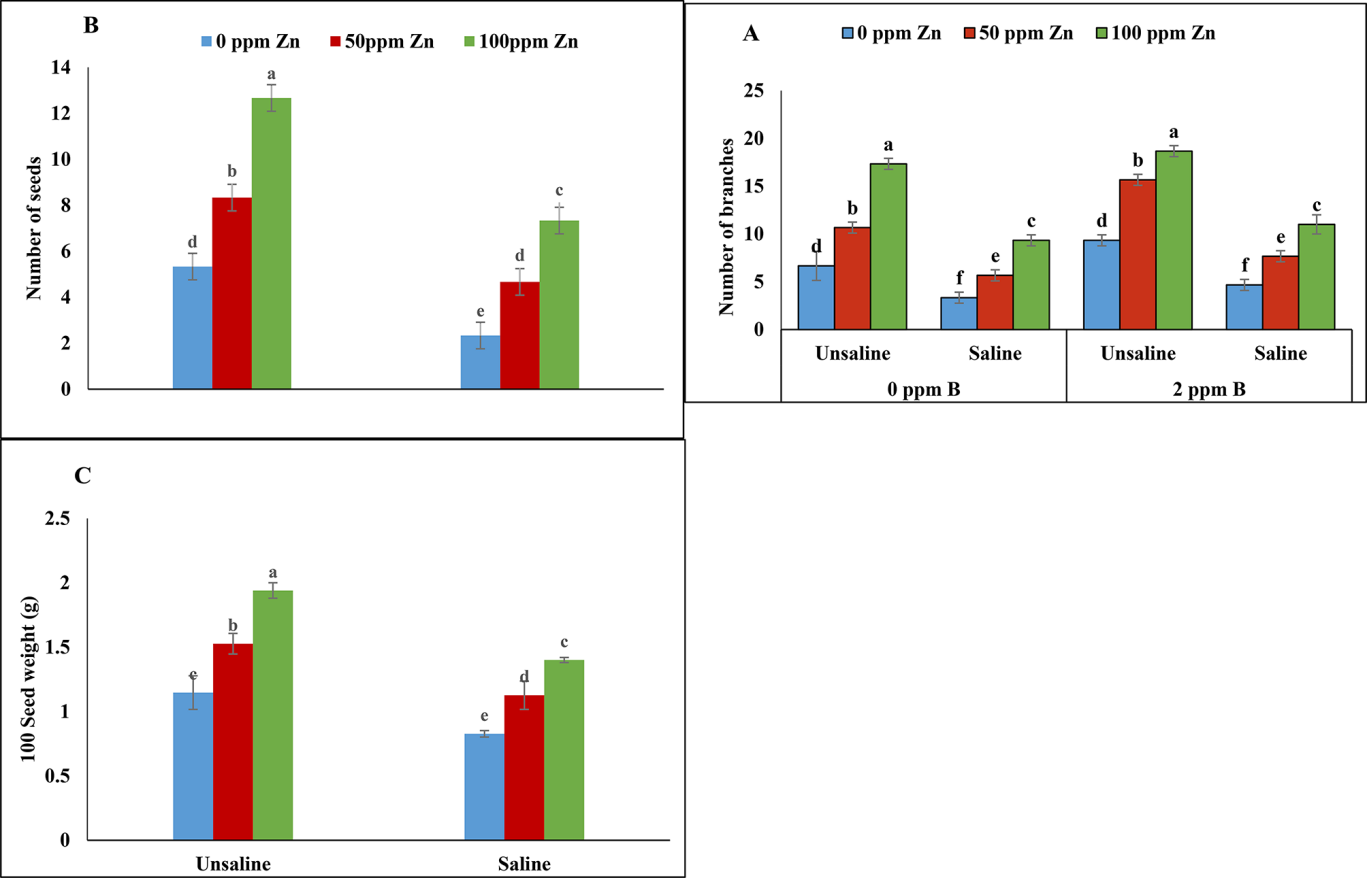
Effect of foliar application of zinc and boron on number of branches **(A)**, number of seeds/pod **(B)** and 100 seed weight **(C)** of fenugreek grown under salinity stress. Values represent means ± S.E. Significant differences among row spacing were measured by the least significant difference (LSD) at *p* > 0.05 and indicated by different letters

According to analysis of variance nutrient analysis for shoot sodium, potassium and calcium ions showed non-significant results under salinity and micronutrient application (zinc and boron). Salinity increased sodium ions and reduced calcium, potassium ions concentration in shoots. Minimum calcium and potassium ions were noted at zinc (0ppm) and boron (0ppm) under saline condition. Foliar application of zinc (100ppm) and boron (2ppm) increased both calcium and potassium ions under saline and un-saline condition non significantly. Maximum values for calcium and potassium ions were recorded on micronutrients (zinc, boron) application under un-saline conditions. However, zinc and boron application resulted in reduction of sodium ions under saline conditions. Minimum sodium ions concentration was recorded at 0ppm of zinc and 2ppm of boron under un-saline conditions (Table 2; Fig. 7A-C).

### Salt tolerance index

**Fig. 8:**
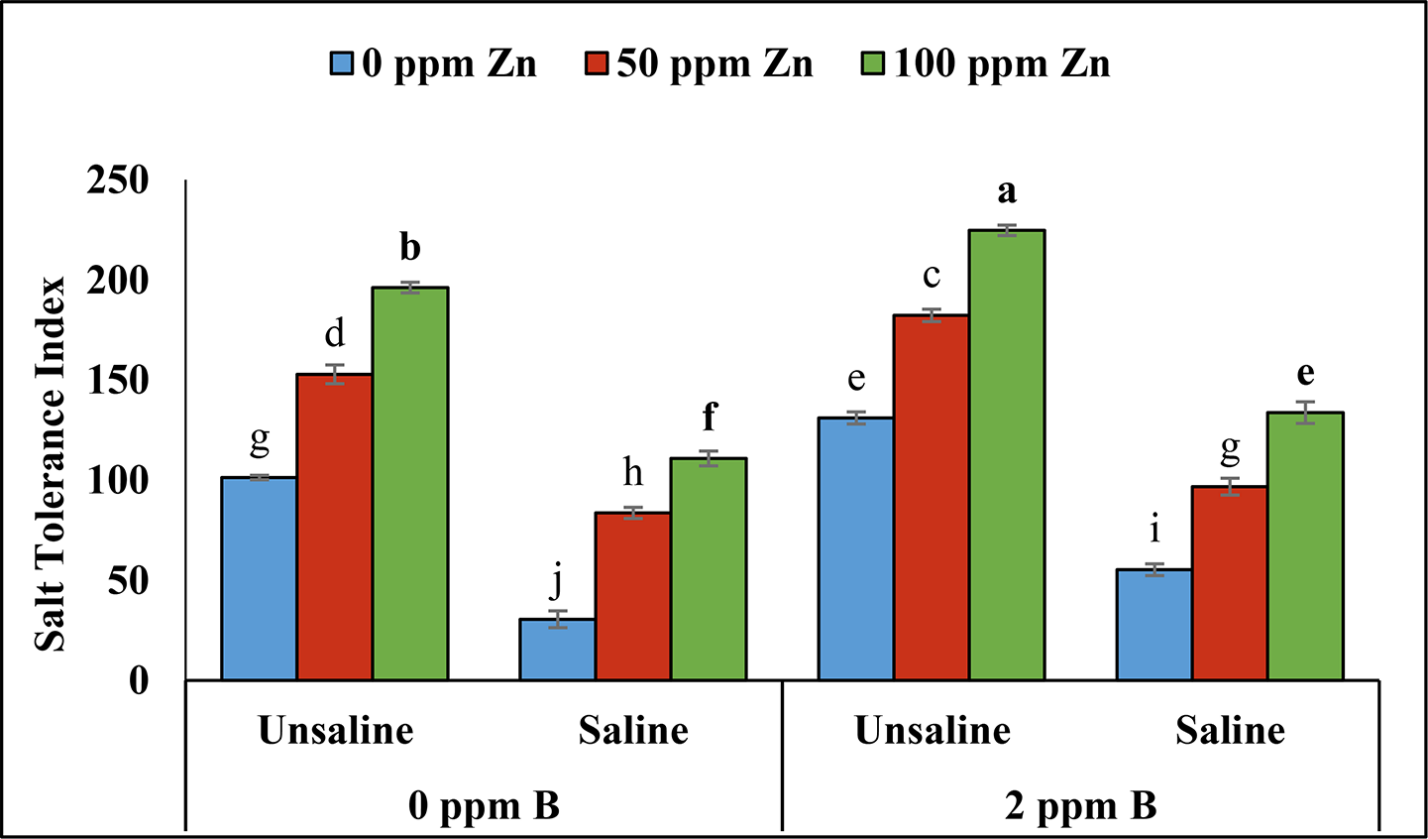
Effect of foliar application of zinc and boron on salt tolerance index of fenugreek grown under salinity stress. Values represent means ± S.E. Significant differences among row spacing were measured by the least significant difference (LSD) at *p* > 0.05 and indicated by different letters

According to the analysis of variance under un-saline conditions salt tolerance index was maximum with foliar application of zinc and boron. However, under saline conditions salt tolerance index was reduced. Minimum salt tolerance was recorded in plants treated with 120mM level of NaCl and control level of zinc and boron. Application of boron (2ppm) and zinc resulted in improving the salt tolerance index of plants under saline conditions (Table 2; Fig. 8).

### Pearson Correlation analysis

**Fig. 9:**
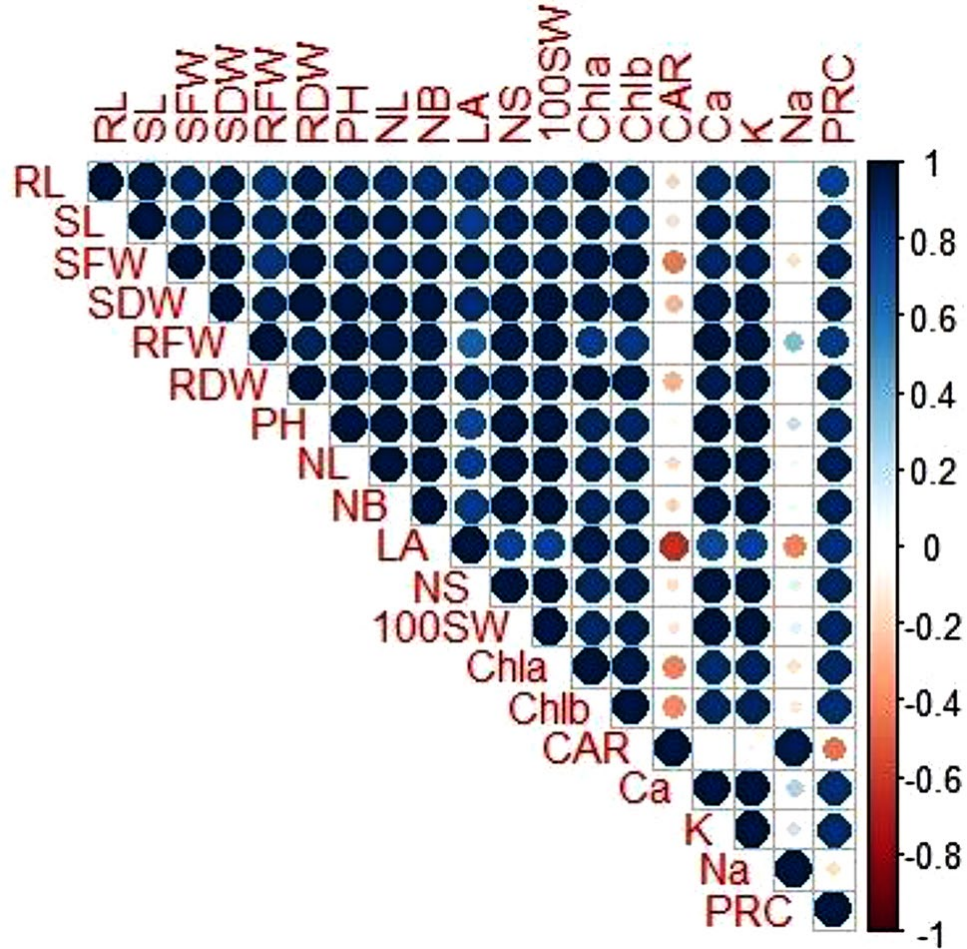
Pearson correlation coefficient representing various growth, physiology and ionic parameters of fenugreek grown under salinity stress with foliar application of zinc and boron. Morphological attributes i.e., root length (RL), shoot length (SL), shoot fresh weight (SFW), root fresh weight (RFW), shoot dry weight (SDW), root dry weight (RDW), leaf area (LA). Physiological attributes: Pigment contents (Cha, Chb, CAR), total soluble proteins (PRC), ionic contents sodium (Na^+^), potassium (K) and calcium (Ca^2+^)

Pearson correlation coefficients for various morpho-physiological attributes are presented in Fig. 9. In this correlation various growth and physiological parameters were correlated under the effect of salinity stress and surfactant treatment with zinc and boron. Root length (RL) strongly correlated with other morphological attributes i.e., shoot height, shoot dry weight (SDW), root dry weight (RDW), physiological at-tributes i.e., chlorophyll a, calcium, and potassium. But protein content (PRC) did not show strong positive correlation whereas shoot length (SL) showed strong correlation with root dry weight (RDW), plant height (PH) and number of leaves (NL). All the morphological data did not show any correlation to sodium (Na^+^) and carotenoids (CAR) content. Calcium (Ca^2+^) and potassium (K) was not linked to sodium (Na^+^), but carotenoids (CAR) content show strong positive correlation with sodium content.

## Discussion

The findings of this research align with prior studies, illustrating the profound impact of environmental conditions on plants. They demonstrate the plants remarkable ability to respond to both biotic [39] and abiotic stresses [40] when subjected to nutrient applications. Among these stresses, salinity stands out as a significant challenge to plant growth and yield [41]. In this study, the deleterious effects of salinity stress on fenugreek plants were evident. This stress resulted in a reduction in fresh and dry weight, root and shoot length, leaf number, and seed production, ultimately leading to decreased plant growth and yield. Micronutrient application, specifically zinc (Zn) and boron (B), emerged as a promising strategy to alleviate the adverse effects of salinity stress on plants. These essential micronutrients play vital roles in plant growth and development [42]. In our present study foliar application of zinc and boron at various levels significantly improved root length, shoot length, and fresh and dry weights under salinity stress.

It is reported that reduction in yield and growth due to salinity stress is attributed to the high concentration of sodium ions, which block the uptake of other essential growth-promoting nutrients [43]. Previous studies reports that micronutrients application play crucial roles in various physiological and biochemical processes within plants, including ion uptake and transport [44]. In this study, the foliar application of zinc and boron enhanced growth and yield of fenugreek plants by reducing sodium ions concentrations. The plant roots play a key role in improving growth and development because roots are important for uptake of water and minerals [45, 46]. Furthermore, the growth of roots is intrinsically linked to potassium ions, which are essential for supporting plants at different growth stages [47–49]. Current study showed that root growth was notably hindered because of elevated salinity stress, resulting in reduced potassium uptake. However, the application of zinc and boron significantly increased the levels of potassium ions, leading to improvement in root growth of plants.

Salinity stress adversely affects photosynthesis, primarily by degrading vital photosynthetic pigments such as chlorophyll a, chlorophyll b, and carotenoids [50]. In this study higher concentration of salinity has reduced the photosynthetic activity which resulted in retarded growth and production of plants. Zinc and boron, being essential for chlorophyll synthesis and efficient photosynthetic activity [51], contributed to mitigating this effect. Their foliar application reduced sodium ions, increased chlorophyll levels, and improved photosynthesis.

Salinity stress is known to disrupt photosynthetic pigments and alter secondary metabolites [52]. In this study, it negatively affected chlorophyll components like chlorophyll a, chlorophyll b, and carotenoids. However, the foliar application of zinc and boron counteracted these effects, enhancing chlorophyll components, reducing salinity stress, and improving photosynthesis.

Salinity, often associated with sodium and chlorine ions, can influence the uptake of calcium and potassium ions in plants [53]. However, the application of micronutrients has been documented to have a beneficial impact on the absorption of these ions, especially in situations where plants are exposed to salinity stress [54]. In our study, zinc and boron were found to positively affect the absorption of these ions under salinity stress. Under saline conditions, sodium ions inhibited the uptake of calcium and potassium ions, but foliar spraying of zinc and boron ameliorated this by enhancing ion levels.

Furthermore, salinity stress had a detrimental impact on the total soluble protein content of the plants. Previous research has indicated that salinity reduces total protein content by diminishing potassium ion concentrations, as the formation of soluble proteins is followed by the availability of potassium ions [55]. In the present study total soluble protein content was decreased under salinity and foliar application zinc with boron enhanced total soluble proteins formation by increasing the potassium ions concentration and reducing Na^+^ ions.

In case of salt tolerance index it is reported from previous literature that salinity tolerance index was reduced with increase of salt stress [56]. In our study it is also noted that under saline conditions the salinity tolerance index was reduced in plants. However, foliar application of zinc and boron improved the salt tolerance index in fenugreek plants.

## Conclusion

Foliar application of zinc and boron proved effective in improving growth and physiological attributes of fenugreek by mitigating salinity stress. Among treatments applied, foliar spray of Zn (100ppm) and B (2ppm) were more effective and showed improvement in growth by enhancing photosynthetic pigments content, root length and shoot length. Foliar spray of B and Zn is recommended to mitigate salinity stress in other crops. In future, this study can be completed by treatment with ZnO NPs instead of Zinc salt application for control release of Zinc. So, this could be a decent nanofertilizer for fenugreek plant. Moreover, molecular basis of this experiment can be tested in future which is lacked in this research. Zinc and boron can also be applied in root medium for other crops to reduce salinity. These micronutrients application can also be tested on other varieties of fenugreek.

## Data Availability

All data generated or analysed during this study are included in this published article.
